# The Chinese medicine JC-001 enhances the chemosensitivity of Lewis lung tumors to cisplatin by modulating the immune response

**DOI:** 10.1186/s12906-017-1728-x

**Published:** 2017-04-11

**Authors:** Meng-Hsien Chuang, Ming-Shiou Jan, Jinghua Tsai Chang, Fung-Jou Lu

**Affiliations:** 1 Institute of Medicine, Chung Shan Medical University, No. 110, Sec. 1, Jianguo N. Rd, Taichung City, 40201 Taiwan; 2 Institute of Biochemistry, Microbiology and Immunology, Chung Shan Medical University, No.110, Sec.1, Jianguo N. Rd, Taichung City, 40201 Taiwan; 3 Immunology Research Center, Chung Shan Medical University, No. 110, Sec. 1, Jianguo N. Rd, Taichung City, 40201 Taiwan; 4 Division of Allergy, Immunology and Rheumatology, Department of Internal Medicine, Chung Shan Medical University Hospital, 110, Sec.1, Jianguo N. Rd, Taichung City, 40201 Taiwan; 5 Department of Chest Medicine, Chung Shan Medical University Hospital, No. 110, Sec. 1, Jianguo N. Rd, Taichung City, 40201 Taiwan; 6 Department of Medical Research, Chung Shan Medical University Hospital, No. 110, Sec. 1, Jianguo N. Rd, Taichung City, 40201 Taiwan

**Keywords:** CDDP, Lung cancer, Immune, Chemosensitivity

## Abstract

**Background:**

JC-001 is a Chinese medicine that can modulate the immunity in Hepa 1-6 tumor-bearing mice, and we questioned whether JC-001 can serve as efficient adjuvant chemotherapy. We aimed to identify a novel approach for enhancing *cis*-diamminedichloroplatinum (II) (CDDP)-based chemotherapy by immunomodulation.

**Methods:**

The anti-tumor activity in vitro was determined based on foci formation and a 3-(4,5-dimethylthiazol-2-yl)-2,5-diphenyltetrazolium bromide (MTT) assay. A LLC1 tumor xenograft model was used to analyze the activity of tumor rejection in vivo. The tumors were analyzed through hematoxylin and eosin (H&E) staining, immunohistochemistry (IHC) staining and cytokine arrays.

**Results:**

JC-001 suppressed foci formation and reduced the viability of Lewis lung carcinoma (LLC1) cells in vitro. JC-001 suppressed LLC1 tumor growth in immunodeficient BALB/c nude mice and in immunocompetent C57BL/6 mice to an even greater extent. Furthermore, JC-001 up-regulated interferon-γ expression in the tumor microenvironment, enhanced the Th1 response in tumor-bearing mice, and increased the chemosensitivity of LLC1 tumors to CDDP chemotherapy. The results of our study suggest that JC-001 is associated with low cytotoxicity and can significantly suppress tumor growth by enhancing the Th1 response.

**Conclusion:**

JC-001 is a Chinese medicine with potential clinical applications in CDDP-based chemotherapeutic regimens.

**Electronic supplementary material:**

The online version of this article (doi:10.1186/s12906-017-1728-x) contains supplementary material, which is available to authorized users.

## Background

Lung cancer is the leading cause of cancer-related deaths worldwide [1]. Lung cancer is generally classified into 2 major types, with 15% of cases classified as small-cell lung cancer (SCLC) and 85% of cases classified as non-small-cell lung cancer (NSCLS). NSCLC can be further divided into the adenocarcinoma (ADCA), squamous cell carcinoma (SCCA), large-cell carcinoma and bronchoalveolar cell carcinoma subtypes [2]. Contributing factors to the pathogenesis of lung cancer include tobacco smoke, genetic aberrations, hormonal factors, chronic obstructive pulmonary disease, and viral infection (human papillomavirus), which ultimately induce tissue damage and changes to the microenvironment [3–6]. Current first-line therapies usually comprise a combination of chemotherapies and targeted therapies directed towards 1 or more of the various signaling pathways implicated in cancer. However, the 5-year survival rate of lung cancer is less than 20% [1]; therefore, more effective therapies are needed.

*cis*-diamminedichloroplatinum (II) (cisplatin; CDDP) is an inorganic compound that has been used to treat malignant tumors over the last 4 decades. However, the side effects of CDDP, including vomiting, digestive tract disorders, ototoxicity and nephrotoxicity, limit its clinical applications [7]. Notably, nephrotoxicity is the primary dose-limiting side effect, as CDDP accumulates in the kidney where it can cause excessive injury to the proximal renal tubule cells [8]. To improve the therapeutic efficacy and minimize toxicity, CDDP is often combined with other chemotherapies or targeted agents. However, the clinical outcomes and quality of life of patients receiving CDDP remains far from ideal [9–11].

In a previous study, we demonstrated that JC-001 suppresses Hepa 1-6 murine tumor cell growth in C57BL/6 mice by modulating the immune response and up-regulates Hepa 1-6-specific cytotoxicity in Hepa 1-6 cells co-cultured with Hepa 1-6-immunized splenocytes [12]. In this study, we evaluated whether JC-001 can suppress the growth of Lewis lung carcinoma cell line (LLC1)-derived tumors and increase the efficacy of CDDP by enhancing its immunomodulatory effects.

## Methods

### Antibodies, chemicals and reagents

Anti-mouse CD8 (BS-0648R) and anti-IFN-γ (BS-0480R) antibodies were purchased from Bioss (Boston, MA, United States). The anti-IL-12 p70 antibody (NBP1-85564) was purchased from Novus Biological (Littleton, MA, United States). Dulbecco’s Modified Eagle Medium (DMEM) culture medium, Roswell Park Memorial Institute (RPMI) medium, L-glutamine, trypsin-EDTA and fetal bovine serum were purchased from Gibco (Grand Island, NY, United States). ELISPOT assay kits for mouse IL-2, IL-10, IL-17A, TNF-α, TGF-β and IFN-γ were purchased from eBioscience (San Diego, CA, United States). Cisplatin was purchased from Sigma-Aldrich (Stockholm, Sweden). Chloral hydrate was purchased from Sigma-Aldrich (Stockholm, Sweden). QuantiChrom™ Creatinine Assay Kit, QuantiChrom™ Urea Assay Kit, EnzyChrom™ Alanine Transaminase Assay Kit, EnzyChrom™ Aspartate Transaminase Assay Kit and QuantiChrom™ BCG Albumin Assay Kit were purchased from BioAssay Systems (Hayward, CA, United States).

### Extraction of plant materials

The Chinese herbal medicine JC-001 was provided by Jun Chen Biotech Co., Ltd., Taiwan. The primary components of this concoction are *Bupleurum chinense* DC (8%), *Gentiana scabra* Bge. (16%), *Rheum palmatum* L. (8%), *Clematis montana* Buch.-Ham. (12%), *Carthamus tinctorius* L. (8%), *Prunus persica* (L.) Batsch (8%), *Angelica dahurica* (Fisch. ex Hoffm.) Benth. et Hook. f. (8%)*, Siegesbeckia orientalis* L. (8%), *Glycyrrhiza uralensis* Fisch. (8%). and *Solanum incanum* L. (16%)*.* The JC-001 hot water extraction process followed our previously described protocol [12].

### Animals and xenograft tumor model

In this study, we followed the guidelines of the Institutional Animal Care and Use Committee (IACUC) of Chung Shan Medical University (CSMU) for the care and use of experimental animals. Eight-week-old female C57BL/6 and male BALB/c nude mice weighing 20-25 g were obtained from BioLASCO (Taichung, Taiwan). The mice were maintained in a 12-h light/dark cycle. After 1 week of adaptation, the experimental procedures were initiated.

In total, 10^5^ LLC1 (ATCC number: CRL-1642; obtained from the Bioresource Collection and Research Center, Taiwan) cells were injected subcutaneously in each eight-week-old female C57BL/6 mouse. After three days, the mice were divided into control (*n* = 15), 1X JC-001 (740 mg/kg; *n* = 14) and 3X JC-001 (2467 mg/kg; *n* = 13) groups and fed with 200 μL of JC-001 or autoclaved double-distilled water daily. At day 23 after tumor inoculation, the mice were anesthetized with a single intraperitoneal injection of 400 mg/kg chloral hydrate, and the tumors were surgically excised, imaged, weighed and fixed in 10% formalin.

### BALB/c nude (immunodeficient) mouse xenograft model

Seven-week-old immunodeficient BALB/c nude mice were housed in individual ventilated cages for 1 week prior to the injection of cancer cells. To prepare the cells for inoculation, 100 μL of RPMI containing 5 × 10^5^ LLC1 cells was combined with 100 μL of Matrigel (10 mg/mL). Each mouse was subcutaneously inoculated with 200 μL of cells/Matrigel solution. On the second day after inoculation, the mice were divided into control (*n* = 8) and 3X JC-001 groups (*n* = 8), and fed with 200 μL of 2467 mg/kg JC-001 or autoclaved double-distilled water. On day 26 after injection, the mice were anesthetized, and the tumors were surgically excised, imaged, weighed and fixed in 10% formalin.

### Foci-formation assay

For the foci-formation assay, 200 LLC1 cancer cells were seeded in a 3.5-cm plate and incubated for 24 h in a 37 °C incubator with 5% CO_2_. The medium was discarded and replaced with 3 mL of fresh medium containing 0 (blank), 50, 100, 200, 400 or 800 μg/mL JC-001. The cells were incubated for approximately 7-9 days without disturbance. When the blank (control) cells formed visible colonies, the cells were washed with 1× PBS and fixed with 100% ethanol for 20 min. After removing the ethanol, the cells were stained with 20% Giemsa for 30 min at room temperature. After the cells were rinsed with water, colony morphology was evaluated, and the number of colonies was counted. These experiments were conducted in triplicate.

### Cell viability assay

The cells (2 × 10^4^) were seeded into 24-well dishes with 1 mL of culture medium and incubated overnight to permit cell adhesion. Then, the medium was refreshed with medium containing 0–800 μg/mL JC-001 (*n* = 4 samples at each concentration). After 96 h of treatment, 400 μL of 3-(4,5-dimethylthiazol-2-yl)-2,5-diphenyltetrazolium bromide (MTT) reagent was added to each well at a final concentration of 500 μg/mL. After four hours of reaction, the reagent was removed, and DMSO was added to dissolve the product. Cell viability was determined by comparing the OD_470_ with that of the control group. These experiments were conducted in triplicate.

### Tumor microenvironment analysis

The RayBio® Mouse Cytokine Antibody Array III kit (RayBiotech, Norcross, GA, United States) was used to detect cytokine changes in tumor tissues. The tumor tissues were frozen in liquid nitrogen and then homogenized in 10 mL of lysis buffer at room temperature for 10 min. The homogenized solution was then centrifuged at 16000 rpm at 4 °C for 2 h. The supernatant was transferred to a new tube, and the concentration was adjusted to 1 mg/mL. A volume of 1 mL of the lysate was added to the RayBio® Mouse Cytokine Antibody Array III, and the chip was incubated at 4 °C overnight with gentle shaking. The chip was then washed with wash buffer I 3 times for 10 min each and subsequently washed with wash buffer II 3 times for 10 min each. The chip was incubated with biotinylated primary antibodies. After washing, the chip was incubated with streptavidin-conjugated secondary antibody. The serum was diluted 1:4 in blocking buffer and directly added to the RayBio® Mouse Cytokine Antibody Array III to allow for binding.

### Co-culture condition and cytokine analysis

To generate the LLC1-immunized splenocytes, C57BL/6 mice were immunized by injection (s.c.) with 5 × 10^3^ LLC1 cells at days 0, 7 and 14. After immunization, the mice were sacrificed, and the spleens were removed aseptically using a routine surgical procedure at day 21. Single-cell suspensions were prepared by filtering the spleen tissue through mesh screens. The erythrocytes were lysed, and the cells were cultured in RPMI-1640 supplemented with 10% FBS and 1% penicillin/streptomycin.

LLC1-immunized splenocytes (4 × 10^6^/well) were co-cultured with LLC1 cells (1 × 10^5^/well) in 24-well plates, and 2 ml of RPMI-1640 supplemented with 10% FBS and 1% penicillin/streptomycin was added to each well. After a 48-h incubation, the culture medium was collected, and cytokine secretion was analyzed using the ELISPOT assay. All reagents used in the ELISPOT assay were supplied in the assay kit, and the assay was conducted according to the manufacturer’s instructions. These experiments were conducted in triplicate.

### CDDP and JC-001 combination therapy in immunocompetent LLC1 tumor model

Eight-week-old C57BL/6 female mice were randomly divided into 5 groups: the naïve (*n* = 6), control (*n* = 16), CDDP (*n* = 16), JC-001 (*n* = 16) and JC-001/CDDP (*n* = 16) groups. All mice were subcutaneously injected with 1 × 10^5^ LLC1 cells. Two days after the inoculation, the mice were fed 200 μL of H_2_O or 2467 mg/kg JC-001 for 18 days. On day 3, 6, 9, 12, 15 and 18 after the inoculation, the mice were treated with 1 mg/kg CDDP (i.p.) or normal saline. On day 22, the mice were anesthetized, and the tumors were surgically excised, imaged, weighed and fixed in 10% formalin. Serum levels of creatinine, urea, alanine aminotransferase (ALT), aspartate transaminase (AST) and albumin were determined by AllBio Science Inc. (Taichung, Taiwan).

### Histological and immunohistochemistry analysis

All the histological work by Rapid Science Co, Ltd., Taichung, Taiwan. And the qualitative analyzed by Prof. Chung-Hung Tsai, Department of Pathology, Chung Shan Medical University Hospital. Both Rapid Science Co, Ltd. and Prof. Chung-Hung Tsai were blind for all the sample. Tumor tissues extracted from the tumor-bearing mice were fixed in 4% formaldehyde buffered with phosphate solution (0.1 mol/L; pH 7.4) and processed for paraffin embedding. The paraffin blocks were sliced into 3-μm sections, stained with hematoxylin and eosin (H&E) and examined using light microscopy. For the immunohistochemistry (IHC) analysis, the slides were deparaffinized in xylene and rehydrated in decreasing concentrations of alcohol. Antigen retrieval was conducted by heating the tissues in a microwave. Then, 3% hydrogen peroxide was used to inactivate endogenous peroxidase, and the samples were blocked with 5% bovine serum in PBS. The slides were incubated at 4 °C overnight with the anti-mouse CD8 (1:400), anti-IL-12 p70 (1:100) or anti-IFN-γ (1:200) primary antibodies. After the slides were washed with PBST, they were sequentially incubated with biotinylated goat anti-rabbit IgG secondary antibody, streptavidin-biotin complex, and 3,3′-diaminobenzidine for 20 min at room temperature.

### Statistical analyses

Statistical analyses of the in vitro experimental data were conducted using the two-tailed Student’s t-test for simple comparisons between 2 values, and values are presented as the mean ± standard deviation. For the in vivo experimental data, the unpaired Student’s t-test was used to compare the means between 2 groups. A *p* value <0.05 was considered statistically significant. All the data were used the GraphPad Prism 5.0 to calculate and statistical analyses.

## Results

### JC-001 inhibited foci formation and disrupted cell viability of LLC1 cells

**Fig. 1:**
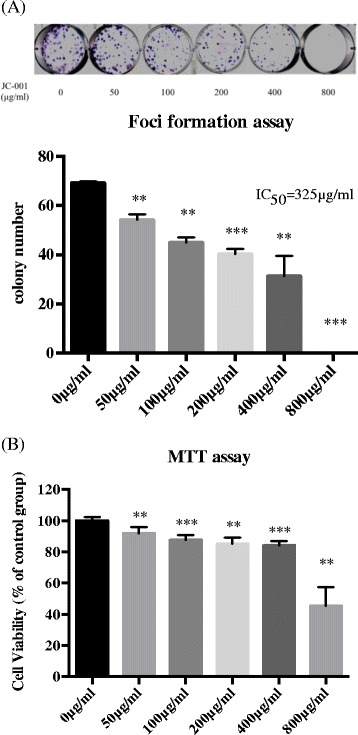
JC-001 affects the foci formation activity and cell viability of LLC1 cells in vitro. **a** The IC_50_ of JC-001 for the inhibition of foci formation by LLC1 was 325 μg/mL. **b** The effect of JC-001 on the cell viability of LLC1. *p < 0.05, **p < 0.01, ***p < 0.001 compared with the control group. The results are expressed as mean ± SD of three separate experiments

To characterize the role of JC-001 in foci formation, LLC1 cells were treated with 0 (blank), 50, 100, 200, 400 or 800 μg/mL JC-001. The number of foci decreased with increasing concentrations of JC-001, with an IC_50_ of 325 μg/mL (Fig. 1a). In addition to the decrease in colony size and number, JC-001 treatment also rendered the colonies less adherent, indicating that JC-001 might affect cell-cell interactions. To determine whether the inhibition of foci formation was due to a disruption of cell viability, LLC1 cells were treated with 0 (blank), 50, 100, 200, 400 or 800 μg/mL JC-001, and the MTT assay was conducted 96 h after the treatment. We found that 0-400 μg/mL JC-001 inhibited cell viability by approximately 10%, whereas JC-001 at a concentration of 800 μg/mL inhibited cell viability by 55% (Fig. 1b). The observation that 100 μg/mL JC-001 inhibited foci formation but not cell viability suggested that JC-001 inhibits foci formation in LLC1 cells in a growth-independent manner.

### The anti-tumor activity of JC-001 in the immunocompetent xenograft tumor model

**Fig. 2:**
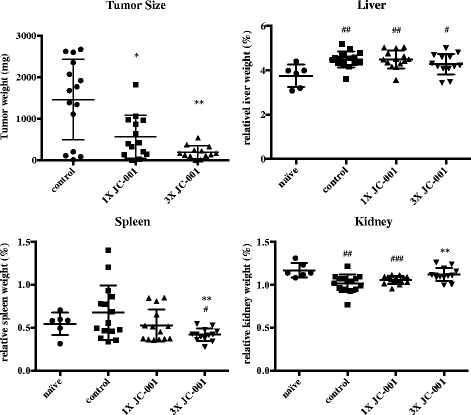
JC-001 inhibits tumor growth and prevents organ damage in LLC1-inoculated C57BL/6 immunocompetent mice. After LLC1 tumor growth for 23 days, the average tumor weight in the control group reached 1461 mg, and the relative organ weights differed from those in the naïve group. The average tumor weight in the 3X JC-001-treated group was significantly reduced to 193 mg. *p < 0.05, **p < 0.01, ***p < 0.001 compared with the control group. #*p* < 0.05, ##*p* < 0.01, ###*p* < 0.001 compared with the naïve group

As JC-001 has the ability to inhibit both foci formation and growth of LLC1 cells, we investigated whether JC-001 exerts anti-tumor activity in an LLC1 xenograft model. The immunocompetent C57BL/6 mice were subcutaneously injected with 1 × 10^5^ LLC1 cells. Three days after the inoculation, the mice were treated with 200 μL of H_2_O, 1X JC-001 (740 mg/kg) or 3X JC-001 (2467 mg/kg) daily. After 20 days of treatment, the tumor mass in mice treated with 1X JC-001 or 3X JC-001 decreased by 59% and 86%, respectively, compared with the H_2_O control group (Fig. 2). In addition, hepatosplenomegaly and renal atrophy were observed in mice inoculated with LLC1 cells, and the severity of these defects was reduced in the JC-001-treated groups compared with the control group. These results indicated that JC-001 effectively inhibited tumor progression in immunocompetent mice.

### The effect of JC-001 on cytokine expression in conditioned medium from a LLC1/splenocyte co-culture

**Fig. 3:**
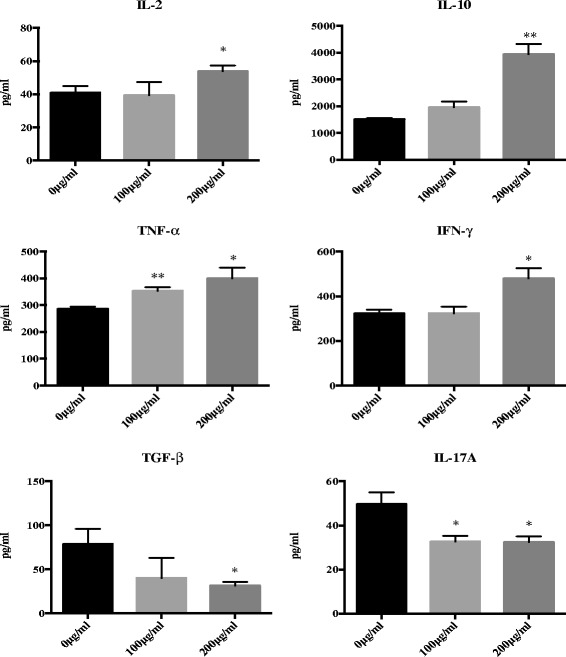
JC-001 mediates cytokine secretion under co-culture conditions. The effector cell (LLC1 immunized splenocytes) to target cell (LLC1 cells) ratio was 40, and each bar represents the mean ± standard deviation (*n* = 3). *p < 0.05, **p < 0.01 compared with the control group. After co-culture for 48 h, JC-001 up-regulated IL-2, IL-10, TNF-α and IFN-γ and down-regulated IL-17A, and TGF-β in the condition medium. The results are expressed as mean ± SD of three separate experiments

To characterize the function of JC-001 in the tumor microenvironment, we immunized C57BL/6 mice with 5 × 10^3^ LLC1 cells to generate LLC1-immunized splenocytes. We co-cultured the effector (LLC1 immunized splenoctyes) and target cells (LLC1 cells) and analyzed cytokine secretion in the culture medium after 48 h of JC-001 treatment. We found that JC-001 up-regulated the expression of IL-2, IL-10, TNF-α, and IFN-γ and down-regulated the expression of TGF-β and IL-17A in the conditioned medium (Fig. 3). The expression patterns of TNF-α, IFN-γ and IL-17 were consistent with the results of the cytokine array assay (Additional file 1) used to evaluate the tumor microenvironment. These findings indicated that JC-001 enhanced theTh1 response and inhibited the Th17 response.

### JC-001 enhanced chemosensitivity to CDDP in an immunocompetent LCC1 xenograft tumor model

The significant nephrotoxicity associated with CDDP limits its clinical applications as an anti-tumor agent. Therefore, we evaluated whether JC-001 treatment could enhance the anti-tumor activity of low-dose CDDP chemotherapy.

**Fig. 4:**
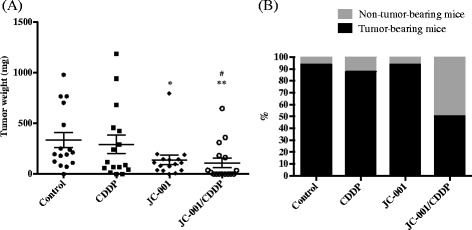
JC-001 enhances the chemosensitivity of the LLC1 tumor model to CDDP. **a** Tumor mass. **b** Percentages of tumor/non-tumor-bearing mice in the groups (*n* = 16). *p < 0.05, **p < 0.01 compared with the control group, #*p* < 0.05 compared with the CDDP-treated group. After JC-001/CDDP combined therapy, the percentage of non-tumor-bearing mice was significantly enhanced from 6.25% to 50% compared with the control group, *p* = 0.0031

**Fig. 5:**
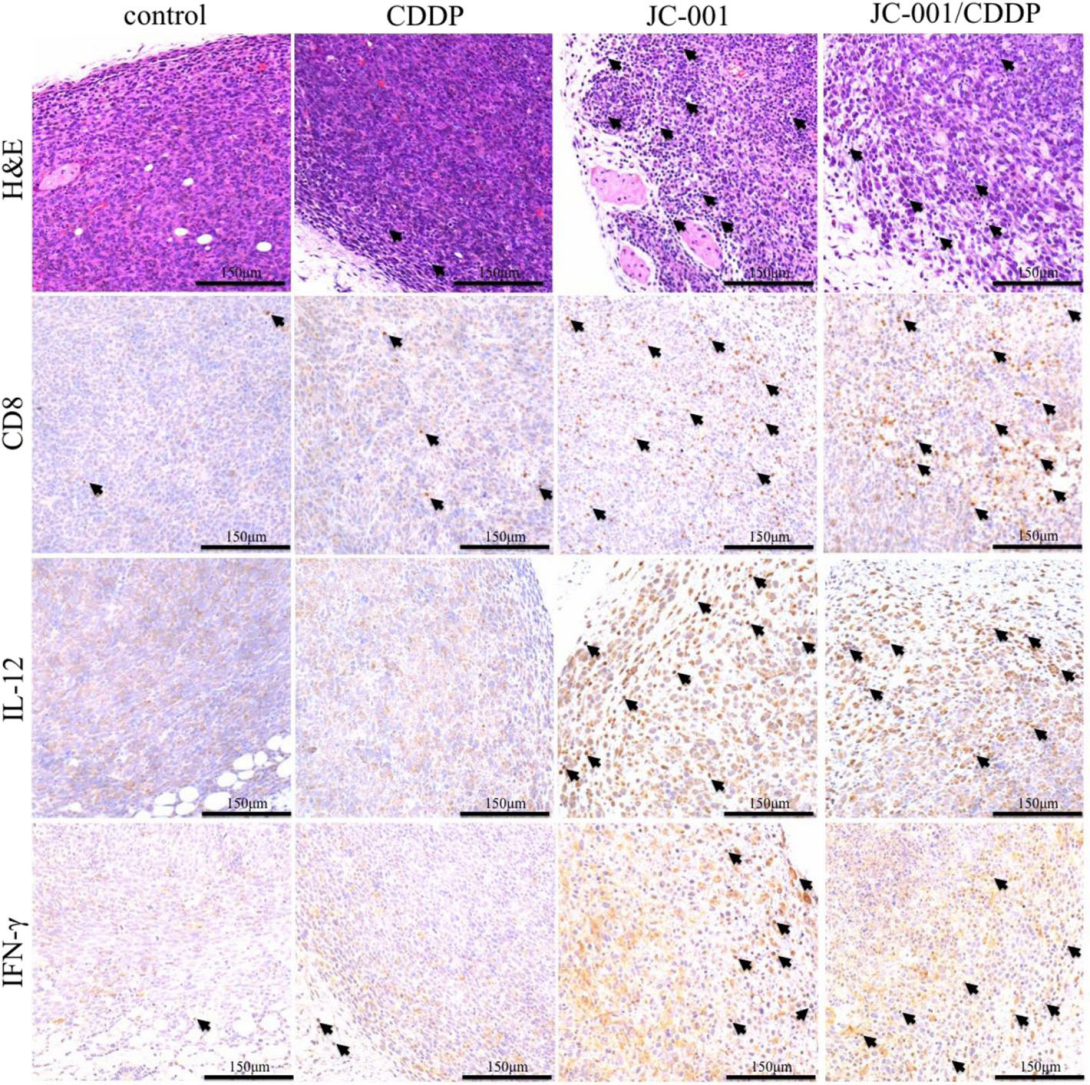
H&E and IHC staining for CD8, IL-12 and IFN-γ in subcutaneous LLC1 tumors. Original magnification: 200×

There were no differences in the average tumor weight in mice injected with 1 mg/kg CDDP six times and the control group. A total of 12.5% of mice in this group did not develop tumors (Fig. 4), which was a slight increase from the 6.25% observed in the control group. The average tumor weight decreased by 41% in the JC-001-treated group compared with the control group, but the percentage of non-tumor-bearing mice was similar to the control group. In mice treated with CDDP in combination with 3X JC-001, a 33% reduction in the average tumor weight was observed, and the percent of non-tumor bearing mice increased to 50%. Serum creatinine, urea, AST and albumin did not differ significantly among the groups. In two mice in the CDDP group and JC-001/CDDP group, serum creatinine reached 6.58 mg/dL and 3.30 mg/dL, respectively (Table 1). In addition, serum ALT in the control group and CDDP group exhibited a significant upward trend compared with the naive group, but this trend was not observed in the JC-001 group and JC-001/CDDP group. Furthermore, an increase in immune cell infiltration was observed in tumor tissues derived from the JC-001- and JC-001/CDDP-treated mice compared with the control group, as demonstrated by H&E staining (Fig. 5). CD8-positive lymphocytes were also observed in tumor tissues derived from JC-001- and JC-001/CDDP-treated mice. IHC staining revealed that the expression of the Th1 cytokine IL-12 p70 and IFN-γ were up-regulated in the JC-001- and JC-001/CDDP-treated tumors. These findings indicated that JC-001 significantly improved the chemosensitivity of LCC1-derived tumors to CDDP by modulating the immune response and successfully reduced tumor formation by 50%.

**Table 1:** Effect of JC-001/CDDP and combined treatment on serum creatinine, urea, ALT, AST and albumin

	Naïve	Control	CDDP	JC-001	JC-001/CDDP
Creatinine (mg/dL)	1.15 ± 0.39	1.27 ± 0.41	1.67 ± 1.48	1.14 ± 0.49	1.44 ± 0.67
Urea (mg/dL)	54.12 ± 9.34	54.79 ± 6.25	52.16 ± 4.05	47.55 ± 9.29	50.79 ± 6.92
ALT (U/L)	37.50 ± 10.19	75.79 ± 50.91*	89.58 ± 62.74*	52.66 ± 29.25	79.06 ± 64.80
AST (U/L)	245.6 ± 23.2	239.8 ± 23.2	238.4 ± 27.9	251.3 ± 21.27	250.5 ± 34.4
Albumin (g/dL)	0.49 ± 0.07	0.46 ± 0.05	0.47 ± 0.04	0.46 ± 0.05	0.47 ± 0.04

*p < 0.05 compared with the naïve group

## Discussion

The murine Lewis lung cancer cell line LLC1 was initially isolated from a spontaneous carcinoma in a C57BL/6 mouse. LLC1 is a fast-growing cell line with a high invasive capability in vitro. We used LLC1 cells to establish xenograft models in both immunocompetent and immunodeficient animals.

In the immunocompetent C57BL/6 xenograft model, JC-001 effectively inhibited LLC1 tumor growth. Treatment with 3X JC-001 (2467 mg/ml) reduced tumor weight by 86% compared with the H_2_O control treatment and prevented tumor formation entirely in 1 mouse. Although only a 50% reduction in tumor mass was observed in the group treated with 10X JC-001 (7400 mg/kg), 3 mice were tumor-free (data not show). As JC-001 inhibited foci formation in LLC1 cells, we sought to determine whether the anti-tumor activity of JC-001 was mediated by the inhibition of LLC1 cell growth or by an enhancement in immunity in C57BL/6 mice.

To further investigate the role of adaptive immunity in JC-001-mediated anti-tumor activity, we established an LLC1 xenograft model in immunodeficient mice (BALB/c nude mice). In mice treated with 3X JC-001, we observed a 61% reduction in tumor mass compared with the H_2_O control group (Additional file 1). Although BALB/c mice are T-cell deficient, they retain functional innate immunity. The 86% reduction in average tumor mass observed in C57BL/6 immunocompetent mice compared with the 61% reduction of tumor mass in BALB/c nude mice suggests that approximately 30% of the anti-tumor activity induced by 3X JC-001 in C57BL/6 mice resulted from the enhancement of the adaptive immune response. The remainder of the effect might have resulted from the enhancement of the innate immune response and/or the inhibition of LLC1 growth. Therefore, we cannot determine whether the anti-tumor activity of JC-001 in BALB/c nude mice was due to inhibiting LLC1 cell growth or due to an enhancement of the innate immune response.

Cytokines in the tumor microenvironment and in serum were further analyzed using the RayBio® Mouse Cytokine Antibody Array III kit (Additional file 2). The expression of inflammatory-associated cytokines, including IL-1α, IL-1β and IL-6, were significantly reduced in the tumor microenvironment of JC-001-treated mice, suggesting that JC-001 might decrease tumor-induced inflammation. JC-001 treatment increased the expression of FasL and CX3CL1 (fractalkine) in the tumor microenvironment and in serum, whereas INF-γ expression was elevated exclusively in the tumor microenvironment.

CX3CL1 can be expressed by cancer cells and endothelial cells. CX3CL1 is expressed as both the membrane-bound and secreted isoforms. CX3CR1, the receptor of CX3CL1, is expressed on the membrane of NK cells, CD14 monocytes, cytotoxic T cells and B cells. Thus, secreted CX3CL1 might act as a chemokine that induces infiltrating immune cells to attack tumor cells. Indeed, high levels of CX3CL1 are associated with a more favorable prognosis in patients with gastric adenocarcinoma and breast carcinoma [13, 14]. IFN-γ enhances NK cell activity, antigen presentation, Th1 cell differentiation, and the activity of macrophages and cytotoxic T cells [15–18]. IFN-γ not only induces cell cycle arrest [19–22] but also enhances class I major histocompatibility complex (MHC-I) expression on the surface of tumor cells, a phenomenon that can promote immune surveillance by triggering CD8^+^ T cell recognition [23]. Moreover, IFN-γ might stimulate cells to express apoptosis-associated genes such as FasL. The results of the cytokine array revealed a cytokine signaling loop in the LLC1 xenograft model. The up-regulation of CX3CL1 in the tumor microenvironment might attract infiltrating IFN-γ-expressing NK cells [18] that, in turn, stimulate the expression of FasL to activate apoptosis in tumor cells. Therefore, JC-001 appears to inhibit tumor progression by modulating the CX3CL1/IFN-γ/FasL signaling pathway.

In addition to activating anti-tumor cytokines, JC-001 inhibited the expression of pro-tumor cytokines, including leptin, LIX (CXCL5) and SCF, in both the tumor microenvironment and in serum. Leptin is an adipokine that plays an important role in energy homeostasis and regulates innate and adaptive responses, thereby modulating the tumor microenvironment [24, 25]. The leptin receptor (LEPR), which is expressed in multiple cancers, stimulates the growth and invasion of activated cancer cells and promotes angiogenesis [26, 27]. In addition, a recent study indicated that leptin promotes cancer stem cell survival [28]. The pro-angiogenic cytokine CXCL5 can also enhance the growth and invasion of cancer cells [29, 30]. High levels of CXCL5 correlate with a poor prognosis in patients with pancreatic cancer, colorectal cancer and hepatocellular carcinoma [31–33]. Thus, serum CXCL5 levels might represent a valuable prognostic factor. SCF and its receptor c-kit play an important role in the growth and survival of lung cancer stem cells [34]. Activation of the SCF/c-kit pathway enhances the growth and invasion of pancreatic cancer and colon cancer [35–37]. SCF induces mast cell infiltration and activation in tumors via mast cell-localized c-kit. The infiltrated mast cells secrete multiple pro-inflammatory factors and inhibit the activation of T cells and NK cells, thereby promoting tumor progression [38, 39]. Previous reports have demonstrated that an anti-SCF antibody enhances the sensitivity of cancer cells to chemotherapeutic agents [40, 41].

The variations in cytokine levels induced by JC-001 treatment suggested that JC-001 exerts its anti-tumor activity by enhancing the expression of anti-tumor cytokines and by reducing the expression of pro-tumor cytokines. Further investigation into the modulation of cytokine levels by JC-001 are needed to gain further insight into the molecular mechanisms underlying JC-001-mediated anti-tumor activity. In addition, such studies might facilitate the development of molecular markers that exhibit similar variations in both the tumor microenvironment and serum and that can help predict a patient’s response to JC-001.

IL-17A is produced by Th17 cells and promotes a pro-tumor environment [42, 43]. In the tumor microenvironment, IL-17A promotes neutrophil recruitment and DNA damage in local tissue by secreting radical oxygen species [44, 45]. We previously reported that JC-001 down-regulates systemic Th17-mediated immunity in both Hepa 1-6 and LLC1 immunocompetent tumor models and up-regulates IL-10 secretion and down-regulates IL-17A in conditioned medium from co-cultured tumor cells and splenocytes. A growing body of evidence indicates that enhancing the immune response can increase the efficacy of chemotherapy [46–48]. The observation that JC-001 modulated the immune response indicates that JC-001 might enhance the clinical applications of CDDP. As the clinical application of CDDP is restricted by dose-limiting toxicity, we evaluated the effect of supplementing low dose CDDP treatment with JC-001. We found that JC-001 inhibited tumor formation by 50% and enhanced the Th1 response in the tumor microenvironment. These outcomes indicate that JC-001 can significantly enhance the anti-tumor effect of CDDP and avoid the toxicity caused by high doses, thereby expanding its potential clinical applications.

## Conclusion

Our data revealed that JC-001 could increase CD8-positive lymphocytes and enhance Th1 response in tumor microenvironment. Moreover, JC-001 significantly improved CDDP chemosensitivity and decreased CDDP toxicity in LLC tumor-bearing mice. These findings suggested that JC-001 is a potential clinical application that may avoid the side effect with high dose CDDP in CDDP-based chemotherapeutic regimens.    

## Additional files

Additional file 1: JC-001 reduced the tumor mass in BALB/c nude immunodeficient mice inoculated with LLC1 subcutaneously. The average tumor weight in the 3X JC-001-treated group (*n* = 8) was significantly reduced to 39% of that in the control group (*n* = 8). **p < 0.01 compared with the control group. (DOCX 67kb)

Additional file 2: Relative cytokine levels in the tumor microenvironment (A) and serum (B) in the JC-001-treated group compared with the control group. LLC1 tumor-bearing mice were treated with 3X JC-001 in H2O for 23 days, and cytokine levels in tumor tissues were analyzed by RayBio®mouse cytokine antibody array 3.1. (DOCX 260 kb)

## Abbreviations

ADCA: Adenocarcinoma
ALT: Alanine aminotransferase
AST: Aspartate transaminase
CDDP: *cis*-diamminedichloroplatinum (II)
CX3CL1: Fractalkine
CXCL5: C-X-C motif chemokine 5
FasL: Fas ligand
H&E: Hematoxylin and eosin
IFN-γ: Interferon gamma
IHC: Immunohistochemistry
IL: Interleukin
MHC-I: Class I major histocompatibility complex
MTT: 3-(4,5-dimethylthiazol-2-yl)-2,5-diphenyltetrazolium bromide
NK: Natural killer
NSCLC: Non-small-cell lung cancer
SCCA: Squamous cell carcinoma
SCF: Stem cell factor
SCLC: Small-cell lung cancer
Th1: T helper type I
Th17: T helper type 17
TNF-α: Tumor necrosis factor alpha
