# Supplementary material for: The Chinese medicine JC-001 enhances the chemosensitivity of Lewis lung tumors to cisplatin by modulating the immune response
Source: BMC Complement Altern Med. 2017 Apr 11;17:210. doi: 10.1186/s12906-017-1728-x (PMC5387375; doi:10.1186/s12906-017-1728-x)
Supplement: Supplementary file 1 — JC-001 reduced the tumor mass in BALB/c nude immunodeficient mice inoculated with LLC1 subcutaneously. The average tumor weight in the 3X JC-001-treated group (n = 8) was significantly reduced to 39% of that in the control group (n = 8). **p < 0.01 compared with the control group. (DOCX 67kb) [file 12906_2017_1728_MOESM1_ESM.docx]

**Additional file 1.** JC-001 reduced the tumor mass in BALB/c nude immunodeficient mice inoculated with LLC1 subcutaneously. The average tumor weight in the 3X JC-001-treated group (n=8) was significantly reduced to 39% of that in the control group (n=8). ***p*<0.01 compared with the control group.
