# Supplementary material for: The Chinese medicine JC-001 enhances the chemosensitivity of Lewis lung tumors to cisplatin by modulating the immune response
Source: BMC Complement Altern Med. 2017 Apr 11;17:210. doi: 10.1186/s12906-017-1728-x (PMC5387375; doi:10.1186/s12906-017-1728-x)
Supplement: Supplementary file 2 — Relative cytokine levels in the tumor microenvironment (A) and serum (B) in the JC-001-treated group compared with the control group. LLC1 tumor-bearing mice were treated with 3X JC-001 in H2O for 23 days, and cytokine levels in tumor tissues were analyzed by RayBio®mouse cytokine antibody array 3.1. (DOCX 260 kb) [file 12906_2017_1728_MOESM2_ESM.docx]

(A)

(B)

**Additional file 2.** Relative cytokine levels in the tumor microenvironment (A) and serum (B) in the JC-001-treated group compared with the control group. LLC1 tumor-bearing mice were treated with 3X JC-001 in H_2_O for 23 days, and cytokine levels in tumor tissues were analyzed by RayBio^®^ mouse cytokine antibody array 3.1.
